# A Modified Method for Studying Behavioral Paradox of Antioxidants and Their Disproportionate Competitive Kinetic Effect to Scavenge the Peroxyl Radical Formation

**DOI:** 10.1155/2014/931581

**Published:** 2014-02-05

**Authors:** Nusrat Masood, Kaneez Fatima, Suaib Luqman

**Affiliations:** ^1^Molecular Bioprospection Department, Biotechnology Division, CSIR-Central Institute of Medicinal and Aromatic Plants, Lucknow 226015, India; ^2^Academy of Scientific and Innovative Research (AcSIR), CSIR-Central Institute of Medicinal and Aromatic Plants, Lucknow 226015, India

## Abstract

We have described a modified method for evaluating inhibitor of peroxyl radicals, a well-recognized and -documented radical involved in cancer initiation and promotion as well as diseases related to oxidative stress and ageing. We are reporting hydrophilic and lipophilic as well as natural and synthetic forms of antioxidants revealing a diversified behaviour to peroxyl radical in a dose-dependent manner (1 nM–10 **μ**M). A simple kinetic model for the competitive oxidation of an indicator molecule (ABTS) and a various antioxidant by a radical (ROO^•^) is described. The influences of both the concentration of antioxidant and duration of reaction (70 min) on the inhibition of the radical cation absorption are taken into account while determining the activity. The induction time of the reaction was also proposed as a parameter enabling determination of antioxidant content by optimizing and introducing other kinetic parameters in 96-well plate assays. The test evidently improves the original PRTC (peroxyl radical trapping capacity) assay in terms of the amount of chemical used, simultaneous tracking, that is, the generation of the radical taking place continually and the kinetic reduction technique (area under curve, peak value, slope, and *V*_max_).

## 1. Introduction

Peroxyl radicals (ROO^•^), the chain carrying analogs of perhydroxyl radical (HOO^•^) where the H atom is replaced by an organic group (R), are formed due to the oxidation of proteins and lipids [1–4]. Activation of neutrophil during oxidative stress related inflammation also produces peroxyl radicals [5, 6]. They are stable (reduction potential [+] 0.77–[+] 1.44 V) and do not dissociate into oxygen because of the stable C–O bond. Its formation depends on the concentration of oxygen and other reactants as well as the hydrophobic/hydrophilic environment in which the reaction occurs [7–9]. These radicals not only occur in a cell but also transpire in aquatic systems (lakes, rivers, streams, and oceans) and in atmosphere (water droplets). Such successive reactions involving free radicals in biological systems lead to many physiological and pathological progressions [10–18].

In study allied to human disease, lipid peroxidation is correlated with peroxyl radical arbitrated reaction [7, 19, 20], induction of DNA damage by superoxide is additively boosted by peroxyl radicals which is implicated in modification of protein, lipid, and DNA cleavage, nevertheless arsenic-mediated ROS generation produces dimethylarsinic peroxyl radicals [7, 21]. The peroxyl radical, besides playing an outstanding character in radicals dependent shabbiness of membranes and proteins, is also fretful in the pathogenesis of a number of human diseases and disorders involving autoxidation of lipids, inactivation of certain enzymes, cleavage of phosphodiester bond resulting in single and double strand breaks, oxidizing thymidine resulting in mutagenic 5-methyl oxidation products, and causing transversion at deoxyguanosine [22–26]. Involvement of peroxyl radicals in carcinogenesis relevant to tumor initiation and promotion is well reported. Investigation related to cancer and redox biology proved that diminutive consideration has been given to peroxyl radicals and family of oxygen centred free radicals [27]. Modest information has been demeanour for peroxyl reactions, in spite of its importance in anticancer irradiation therapy, neurodisorders, ischaemia, and other oxidative stress related diseases and disorders [28].

A number of ways have been utilized for averting the constant expression of peroxyl radicals generated by water soluble 2,2′ azobis (2-amidinopropane) dihydrochloride (AAPH) or lipid soluble 2,2′-azobis 2, 4-dimethylvaleronitrile (AMVN) [28, 29] and the interaction of peroxyl radicals with antioxidants/unknown compounds can be analyzed by an assortment of indicator molecule/model systems as given in Table 1. These reported methods evaluate how the substrate protects a compound from being degraded by peroxyl radicals, using an array of molecules as reactive targets/analysis of end products by different techniques. We employed ABTS as target molecule to assess the reactivity of antioxidants towards AAPH/ABAP-derived peroxyl radical formation in an easy controlled mode at 37°C in aqueous media.

**Figure 1:**
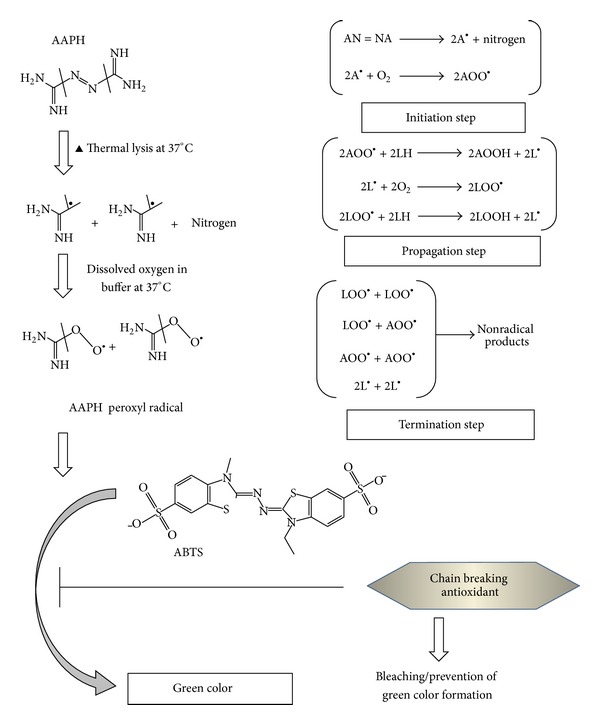
Decomposition of AAPH/ABAP and formation of Peroxyl radicals.

As depicted in Figure 1, there is a unimolecular decomposition of AAPH/ABAP resulting in the formation of two carbon-centred radicals and nitrogen; the former reacts with oxygen to produce peroxyl radicals. We have modified the method of Bartosz et al. (1998) [43] and developed a 96-well plate assay procedure in which decomposition of AAPH (the source of peroxyl and alkoxyl radicals generated at a defined rate in aqueous solution) was measured at 414 nm with ABTS forming a green colored complex. In addition to this juncture, the overall reaction depends on temperature, solvent form, time, and pH, propped up by kinetic studies carried out with the help of spectrophotometric analysis, thus clarifying the paradox behaviour of a series of antioxidants (Table 2). We therefore have a simple high throughput access system and/or technique to identify and explore novel scavenger/inhibitor of peroxyl radicals.

## 2. Materials and Methods

### 2.1. Chemicals

2,2′-Azobis(2-methylpropionamidine) dihydrochloride (AAPH) also known as 2,2′-azobis (2-amido propane) (ABAP), 2,2′azoino-bis(3-ethyl benzothiazoline-6-sulfonic acid) diammoniumsalt (ABTS) 6-hydroxy-2,5,7,8-tetramethylchroman-2-carboxylic acid (trolox), hydrogen peroxide (H_2_O_2_), L-ascorbic acid, and dimethyl sulfoxide (DMSO) were purchased from Sigma Aldrich Chemical Company, USA, while butylated hydroxytoluene (BHT), tert-butylhydroquinone (TBHQ), tert-butyl hydroperoxide (tBHP), butylated hydroxyanisole (BHA), tocopherol, n-propyl gallate, quercetin, and *β*-carotene were purchased from Himedia, India. Di-sodium hydrogen phosphate (Na_2_HPO_4_), sodium dihydrogen phosphate (NaH_2_PO_4_), ethanol, methanol, and acetone were purchased from Merck India Ltd. All the chemicals, solvents and reagents used were either of analytical grade or higher.

### 2.2. Experimental

Stock solution of 0.1 M sodium phosphate buffer (pH 7.0) was made by mixing solutions of Na_2_HPO_4_ (0.1 M) and NaH_2_PO_4_ (0.1 M) while stock solution of ABTS (5 mM) and AAPH (200 mM) was made in deionized water. 10 mM stock solution of tBHP, BHT, L-ascorbic acid, trolox, nicotinic acid, and hydrogen peroxide was made in deionized water, tocopherol, BHA and n-propyl gallate was made in ethanol, TBHQ in methanol, quercetin in DMSO, and *β*-carotene in acetone. Dilution was done in 0.1 M sodium phosphate buffer. The concentrations used for the analysis were 100 *μ*M, 10 *μ*M, 1 *μ*M, 0.1 *μ*M, 0.01 *μ*M, and 0.001 *μ*M.

*Colorimetric peroxyl radical averting assay (PRAA)* was done following method described by Bartosz et al. (1998) [43] with modification including increase in the time period from 10 min to 70 min and reduction in the volume of the reaction cocktail by 20 times. The additional feature of the modified method comprises kinetic reduction technique (area under curve, peak value, slope, time of half maximum, mean, and *V*_max⁡_). Total reaction volume (150 *μ*L) of the modified method in 96-well plate contains 15 *μ*L of samples (10x), 15 *μ*L AAPH (200 mM), 4.5 *μ*L ABTS (5 mM), and rest phosphate buffer. The phosphate buffer was preheated to 37°C for 30 min followed by the addition of other components including AAPH (source of peroxyl radical) added at the last and the absorbance was taken at 414 nm in a kinetic mode by presetting the spectrophotometer at 37°C for 0–70 min. From the kinetic curve, time-dependent increase in the absorbance was read for each compound along with control. Appropriate solvent blank was run in each assay.

### 2.3. Assay Validation, Data Scrutiny, and Statistical Analysis

Data represented are mean/average ± standard deviation of three independent experiments in duplicate. IC_50_ values were calculated from dose-responsive curve by using Table Curve 2D Windows version 4.07 (SPSS Inc., Chicago, IL, USA). Using the advanced kinetic reduction technique available in SoftMax Pro Microplate Data Acquisition and Analysis Software Version 5.3 (Molecular Devices Corporation, Sunnyvale CA, USA) various kinetic parameters were calculated: percent values for *V*_max⁡_ (milli OD/min), peak, slope, and time of half maximum, mean, and area under curve using the formula: ((Control − Unknown)/Control∗100). Pearson and RSQ values were also calculated for different parameters at higher tested concentration.

## 3. Results and Discussion

In the present work, a competitive kinetic method using ABTS as an indicator molecule was employed to estimate the reactivity of antioxidants towards AAPH/ABAP-derived peroxyl radical formation. The results obtained indicate that the relative protection afforded by a given compound strongly depends upon the experimental conditions employed and emphasize the role of secondary reactions of the phenol-derived radicals initially formed. Herein we are reporting hydrophilic and lipophilic as well as natural and synthetic forms of antioxidants revealing a diversified behaviour to peroxyl radical in a dose-dependent manner (1 nM–10 *μ*M). Hydrogen peroxide and tert-butyl hydroperoxide were used as checks. The influence of concentration of the antioxidants, duration of reaction system, and inhibition of the radical (cation or structure of antioxidant) absorption were taken into account while determining the scavenging potential.

**Figure 2:**
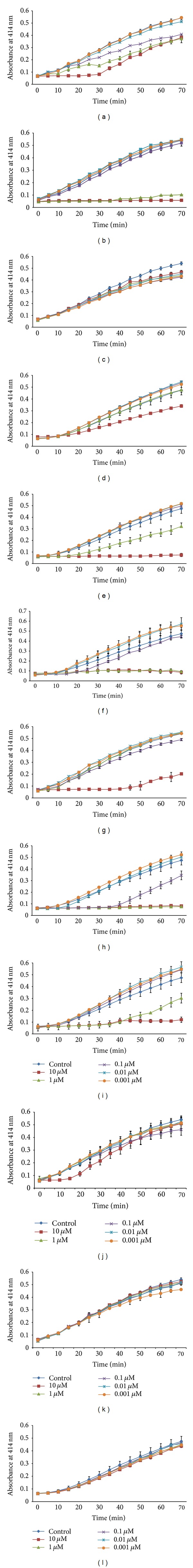
(a) Concentration-dependent peroxyl radical averting ability of trolox. (b) Concentration-dependent peroxyl radical averting ability of *α*-tocopherol. (c) Concentration-dependent peroxyl radical averting ability of nicotinic acid. (d) Concentration-dependent peroxyl radical averting ability of *β*-carotene. (e) Concentration-dependent peroxyl radical averting ability of l-ascorbic acid. (f) Concentration-dependent peroxyl radical averting ability of quercetin. (g) Concentration-dependent peroxyl radical averting ability of n-propyl gallate. (h) Concentration-dependent peroxyl radical averting ability of BHA. (i) Concentration-dependent peroxyl radical averting ability of BHT. (j) Concentration-dependent peroxyl radical averting ability of TBHQ. (k) Concentration-dependent peroxyl radical averting ability of tBHP. (l) Concentration-dependent peroxyl radical averting ability of H_2_O_2_.

Figures 2(a)–2(l) were plotted with control which showed a linear gradient in absorbance (optical density) from 0.06 to 0.6 within 70 min at 414 nm which gradually reaches maximum (0.8–1.0) in 24 h. The time period of 70 min (10 time increase in OD) was chosen in order to get a clear picture of the compound which was not apparent in 10 min incubation time. For IC_50_ calculation, the percent scavenging value of *V*_max⁡_ (milli absorbance/min) was chosen over the lag time period earlier reported to determine the scavenging capacity. It is evident from our plotted curves that not only lag time period but other features should also be considered while evaluating the ability of a particular compound as an effective scavenger of peroxyl radicals. The behaviour of the compounds varies which can be manifested by the graph not following the pattern of the control and the echelon of the off-track hints the individual ability of both concentration and time-dependent.

A comprehensible concentration-dependent (1 nM–10 *μ*M) diminishing interaction with peroxyl radical was observed in trolox (Figure 2(a)). Maximum kinetic inhibition was detected at 10 *μ*M and percent inhibition (in terms of *V*_max⁡_, milli absorbance/min) was found to be in the range of −8.2 to 67.2 proving it as an effective scavenger of peroxyl radical formation in a dose-dependent manner. Trolox, a cell-permeable, water soluble imitative of vitamin E with compelling antioxidant assets [44, 45], is frequently used as a standard or positive control in antioxidant assays. It is also used to gauge the job of oxidative injury in cell death and ageing [46, 47] and as an effective therapy in the treatment of certain cancers [48]. Noteworthy effect was monitored in *α*-tocopherol (Figure 2(b)) at 10 *μ*M and 1 *μ*M concentration. Percent inhibition was found to be in the range of −8.0 to 81.5 compared to control. *α*-Tocopherol is the most imperative (90%) among eight natural tocopherol, as peroxyl radicals scavenger/repressor of lipid peroxidation. Mechanistic study reveals that hydrogen atom is abstracted from the OH group in *α*-tocopherol by a lipid peroxyl radical [AOO^•^] producing fairly inert tocopheroxyl radical [TocO^•^], which may then react with a second radical [AOO^•^] to yield a nonradical product, AOO-Toc, thus destroying two radicals and terminating the radical chain reactions thereby contributing two electrons as a chain breaking antioxidant [49]. Inspite of scavenging peroxyl radicals, they are unable to act as a potent scavenger of hydroxyl, alkoxyl, nitrogen dioxide, and thiyl radicals *in vivo* [50]. Behaviour of nicotinic acid (NA) was uncanny till 40 min and afterwards trifling scavenging started with time (Figure 2(c)). Percent inhibition was found to be in the range of −6.1 to 37.4 as the concentration increases from 1 nM to 10 *μ*M. NA, a colorless, water soluble derivative of pyridine with a carboxyl group at the 3-position, is a cofactor for NAD and NADP acting as coenzymes for more than thousand hydrogenases involved in almost every aspect of cell metabolism [51, 52]. Additionally, it also reduces LDL, VLDL-C, and triglycerides but effectively increases HDL [53] and affects vascular endothelial oxidative and inflammatory events [54]. Much litigious *β*-carotene showed good scavenging effect at 10 *μ*M while at other concentrations the effect was equivalent to basal values (Figure 2(d)). Percent inhibition was found to be in the range of −4.4 to 40.5. Belonging to carotenoid family, *β*-carotene also known as provitamin A has an unsaturated and long aliphatic hydrocarbon chain ultimately splitting into two molecules of vitamin A. Unlike phenolic antioxidants, they do not have reactive hydrogen to contribute to radicals, which make it difficult to use conventional probes for the assessment of their radical scavenging capacity [55]. Earlier investigators [56, 57] observed that it inhibits peroxyl radical-initiated autoxidation of both tetralin and methyl linoleate and it is more effective antioxidant at 15 torr oxygen concentration than at 150 torr. Others have noticed a reticent increase in its action against liposomes at 15 torr [56, 58] and at about 4 torr in microsomes [56, 59]. *β*-Carotene eventually form a resonance-stabilized, carbon-centered radical adduct [AOO-*β*-C^•^]. Interaction of second peroxyl radical to the adduct produces a nonradical product and results in an overall trapping of two peroxyl radicals per *β*-carotene consumed [56]. L-Ascorbic acid was found as an excellent scavenger at 10 *μ*M and 1 *μ*M, an effect which decreases at lower concentrations (Figure 2(e)). Percent inhibition was found to be in the range of −2.5 to 97.7. Also known as vitamin C, ascorbic acid is an important water soluble antioxidant in extracellular fluid present in its deprotonated state under most physiologic conditions [60–62]. It is effective scavenger of superoxide anion radical, hydrogen peroxide, hypochlorite, the hydroxyl radical, and peroxyl radicals [60, 63–69]. Comparatively, it is more effective in inhibiting lipid peroxidation initiated by a peroxyl radical than other human plasma components, such as protein thiols, urate, bilirubin, and *α*-tocopherol [60, 70]. Ascorbic acid can also protect membranes against peroxidation by enhancing the action of tocopherol and thereby reestablishing the radical scavenging activity [60, 71–74]. Quercetin showed a preeminent scavenging of peroxyl radical formation both at 10 *μ*M and 1 *μ*M concentration (Figure 2(f)) and the percent scavenging effect was found to be in the range of 11.48 to 98.3 in a concentration-dependent manner. It is a flavonoid phytochemical naturally occurring in the rind and bark of numerous plants. Chemically it is 2-(3,4-dihydroxyphenyl)-3,5,7-trihydroxy-4H-1-benzopyran-4-one where B-ring is the active center for scavenging and stabilizing the free radicals [75–77]. Best effect of averting action of peroxyl radical by n-propyl gallate was detected at 10 *μ*M with marginal to no scavenging was found at 0.1 *μ*M, 1 *μ*M, 10 nM, and 1 nM concentration (Figure 2(g)). Percent averting action was found to be in the range of 17.17 to 79.57. Propyl gallate, obtained from natural gallic acid (3,4,5-trihydroxybenzoic acid, C_6_H_2_(OH)_3_COOH), is one of the most effective antioxidant-based antimicrobials for the food industry. It has two functional groups hydroxyl and carboxyl and its two analogues were more effective than trolox in preventing cell lysis of human erythrocytes induced by peroxyl radical initiator [78]. Butylated hydroxyanisole (BHA) and butyl hydroxytoluene (BHT) are synthetic antioxidants exhibiting diverse effect. Significant inhibition was observed at 10 *μ*M and 1 *μ*M which decreases at lower dose (Figures 2(h) and 2(i)). Percent inhibition was found to be in the range of 23.75 to 96.6 and −10.9 to 96.2 for BHA and BHT, respectively. BHA consists of a mixture of two isomeric organic compounds, 2-*tert*-butyl-4-hydroxyanisole and 3-*tert*-butyl-4-hydroxyanisole. It is a waxy solid used as a food additive (E320), as an antioxidant and preservative in food, food packaging, animal feed, cosmetics, rubber, and petroleum products to prevent from rancidity and developing objectionable odors. It is also used in medicines, such as isotretinoin, lovastatin, and simvastatin [79]. BHT is a lipophilic organic compound which behaves as a synthetic analogue of vitamin E, primarily acting as a terminating agent that suppresses autoxidation by converting peroxyl radicals to hydroperoxides through donating a hydrogen atom [80]. EC_50_ value for BHA and BHT was found to be 18.94 ± 0.38 and 182.69 ± 13.7 *μ*M, respectively, in an ABAP generated peroxyl radicals, measured by inhibition of dichlorofluorescein oxidation [33]. Like BHT, the conjugated aromatic ring of BHA is able to stabilize free radicals through sequestration. Tertiary butylhydroquinone (TBHQ) flaunted good forestalling action at 10 *μ*M while fairly negligible effects were seen at other concentration (Figure 2(j)). Percent inhibition was found to be in the range of 19.97 to 61.37 in a concentration-dependent manner. TBHQ, a derivative of hydroquinone, substituted with *tert*-butyl group reacts with peroxyl radicals to form a semiquinone resonance hybrid which undergo different reactions to form more stable products reacting with one another to form dimers, dismutate, and regenerate as semiquinones before finally counteracting with another peroxy radical [81]. Interaction with peroxyl radicals exhibited no effect with *tert*-butyl hydroperoxide (Figure 2(k)) and hydrogen peroxide (Figure 2(l)). Both *tert*-butyl hydroperoxide (tBuOOH/tBHP) and hydrogen peroxide (H_2_O_2_) were used as check compound for seeing the effect in peroxyl radical formation. tBuOOH/tBHP used in a variety of oxidation processes depletes GSH, induces lipid peroxidation, and tempts ROS formation, involved in PLA(2) activation in hepatocyte injury [82], responsible for K^+^ leakage [83]. Hydrogen peroxide (H_2_O_2_) is a potent oxidant and is even more toxic to cells than superoxide radicals removed by enzymes, such as catalase, glutathione peroxidases, and cysteinyl peroxidase [49].

**Figure 3:**
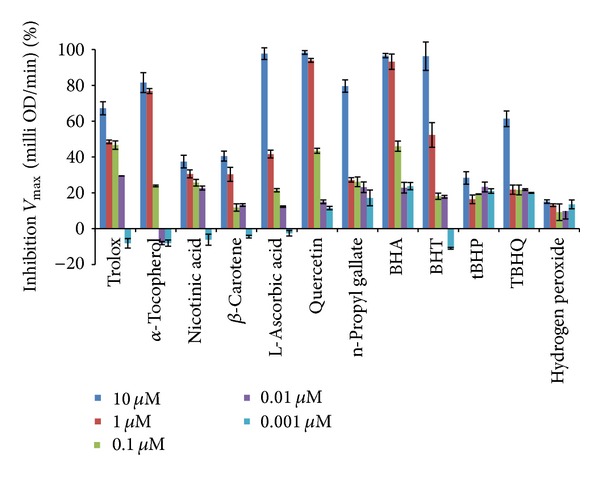
Percent inhibition calculated on the basis of *V*_max⁡_ (milliOD/min) of the kinetic curve.

Antioxidants deactivate free radicals either by reduction via electron or by hydrogen atom. The end point result is the same regardless of the mechanism but the kinetics differ. For kinetic analysis we have used software in which kinetic data reduction options were present. We tried different options present to get a vivid picture of our experimental observations. All the kinetic parameters were calculated with respect to control as percent inhibition. The advantage of calculating using percent inhibition brought all kinetic parameters on the same platform in order to judge the kinetic behaviour characteristics independently. Percent inhibition of *V*_max⁡_ (milli OD/min) of the kinetic curve was plotted for all twelve compounds (Figure 3) and IC_50_ values were calculated from table curve as given in Table 3. *V*_max⁡_ is the maximum slope of the kinetic display of mOD/min, calculated by measuring the slopes of a number of straight lines, where *V*_max⁡_ points determine the number of contiguous points over which each straight line is defined. This is an alternative method for analyzing nonlinear kinetic reactions that reports the elapsed time until the maximum reaction rate is reached, rather than reporting the maximum rate itself. *V*_max⁡_ rate is reported as signal/min (milli-OD/min) for a kinetic read. It is calculated using a linear curve fit, *y* = *Ax* + *B*. A creeping iteration is performed using *V*_max⁡_ points and the slope of the steepest line segment is reported as *V*_max⁡_ rate. The first slope is calculated for a line drawn beginning at the first reading as defined by lag time and ending at a total number of readings equal to the *V*_max⁡_ points setting. The second and any subsequent slopes are calculated beginning at the second time point and ending at a total number of readings. The steepest positive or negative slope is reported as *V*_max⁡_. Decreasing order for the highest concentration used (10 *μ*M) is quercetin > L-ascorbic acid > BHA > BHT > *α*-tocopherol > trolox > TBHQ > *β*-carotene > n-propyl gallate > nicotinic acid > tBHP > H_2_O_2_.

**Figure 4:**
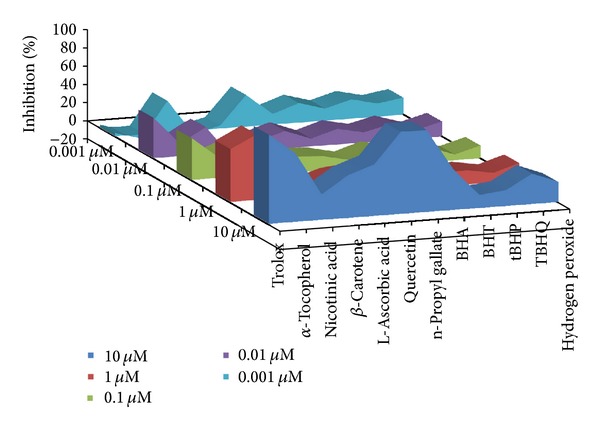
Percent area under curve with respect to control.

**Figure 5:**
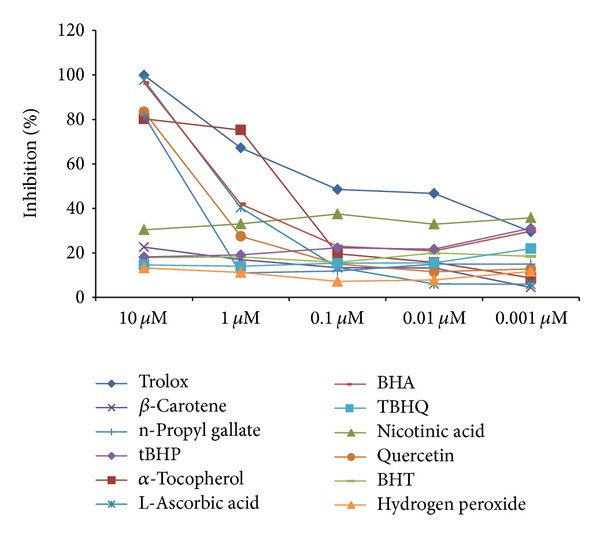
Percent slope (rate) of the time-dependent kinetics with respect to control.

Percent inhibition of area under curve is represented in Figure 4. Defined by the data within the reduction limits, plots are treated as a series of trapezoids with vertices at successive data points and at the *x*-axis coordinates of the data points. The areas defined by each of the trapezoids are then computed and summed. Order was found to be trolox > quercetin > n-propyl gallate > *α*-tocopherol > L-ascorbic acid > *β* carotene > BHA > TBHQ > nicotinic acid > H_2_O_2_ > tBHP > BHT.  Figure 5 signifies the percent slope (rate) of the time-dependent kinetics with respect to control. The slope reduction option determines the slope of the combined plot using all visible time points in the reduction window. Slope is the same as *V*_max⁡_ rate when *V*_max⁡_ rate is set to the same number of points as the run but is different if we have modified *V*_max⁡_ points. Order noticed was trolox > L-ascorbic acid > BHA > quercetin > n-propyl gallate > *α*-tocopherol > nicotinic acid > *β*-carotene > BHT > tBHP > TBHQ > H_2_O_2_.

**Figure 6:**
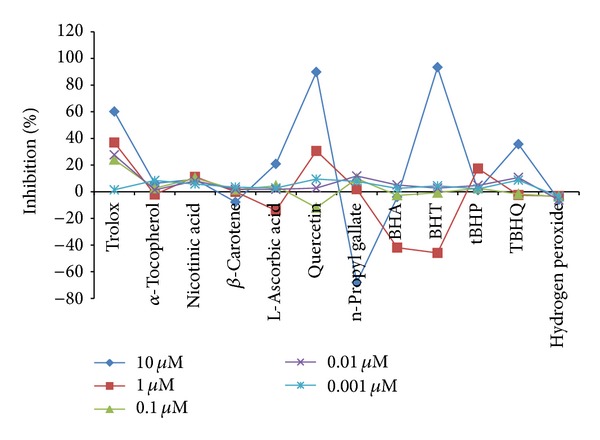
Percent values with respect to control calculated on the basis of time to half maximum of the kinetic curve.

**Figure 7:**
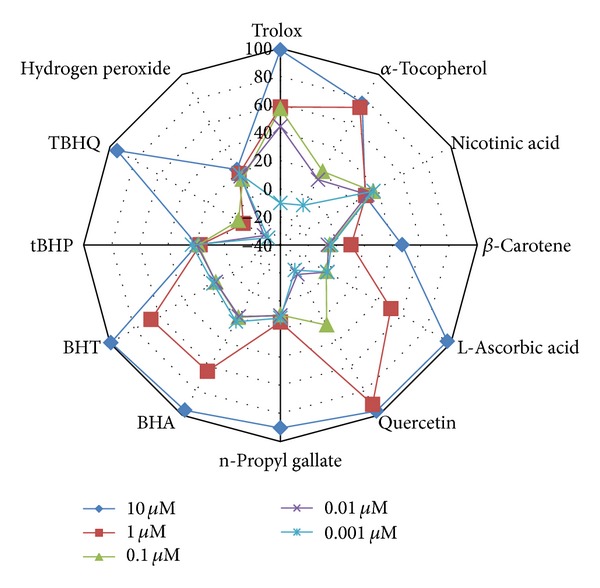
Percent mean values with respect to control. Mean values represent the average values (OD) generated during the time specified.

**Figure 8:**
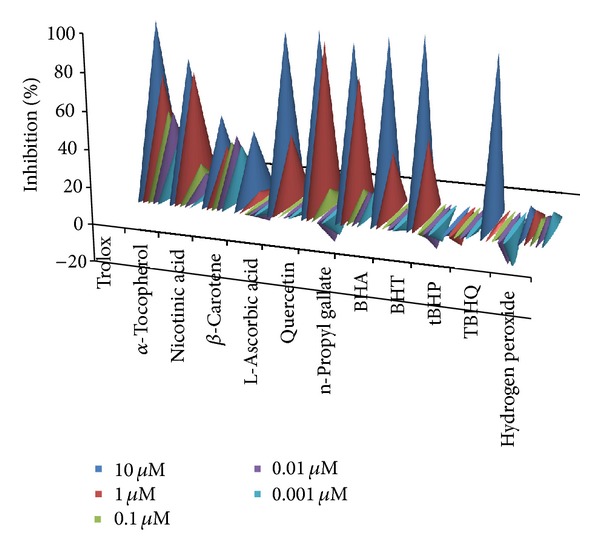
Percent inhibition is calculated from peak values of the kinetic data with respect to control. Peak values represent maximum OD of the compound at this wavelength.

Implication of the percent values with respect to control was also calculated on the basis of time to half maximum of the kinetic curve (Figure 6). It denotes half of maximum OD to the time that falls within the reduction limits. The software determines the kinetic point that has maximum OD, divided by 2 thus getting 1/2 maximum values; further it finds the time at this 1/2 maximum value. Order of the percent values was recorded as BHT > quercetin > trolox > TBHQ > L-ascorbic acid > nicotinic acid > *α*-tocopherol > tBHP > BHA > H_2_O_2_ > *β* carotene > n-propyl gallate.  Figure 7 designates percent mean values with respect to control, representing the average values (OD) generated during the specified time. Order was found to be BHT > trolox > L-ascorbic acid > quercetin > BHA > TBHQ > n-propyl gallate > *α*-tocopherol > *β*-carotene > nicotinic acid > H_2_O_2_ > tBHP.  Figure 8 represents percent inhibition calculated from peak values of the kinetic data with respect to control, representing maximum absorbance of the compound at 414 nm. Order was found to be BHT > trolox > quercetin > L-ascorbic acid > BHA > TBHQ > n-propyl gallate > *α*-tocopherol > nicotinic acid > *β*-carotene > H_2_O_2_ > tBHP. Pearson “*r*” denotes the Pearson product moment correlation coefficient (Table 4). RSQ “*r*2” returns the square of the Pearson product moment correlation coefficient through the given data points. “*r*2” value is interpreted as the proportion of the variance in *y* attributable to the variance in *x*, where *x* and *y* represent different parameters in sequence. “*r*2” is the square of this correlation coefficient presented in Table 5.

## 4. Conclusion

Redox biology, an inescapable field known for its beneficial/detrimental property is being studied extensively. Radicals can wreak devastation on macromolecules/metabolites and may cause short/long term effects on cell signalling. Lipid peroxidation has been the issue of far-reaching scrutiny of mechanistic cell signalling and its involvement in human diseases/disorders. The development of a high-throughput absorbance assay for monitoring kinetics of peroxyl radical reactions in vitro is described in this paper where the evolution of the increase in absorbance values over time provides a rapid, facile method to conduct competitive kinetic studies in the presence of different antioxidants. A quantitative treatment formulated for the temporal evolution of the kinetic interpretation in terms of different parameters is presented. Combined, competitive kinetic assay and the data analysis provides a new method to obtain, in a rapid, parallel format, relative antioxidant capacity to retard the formation of peroxyl radicals. These data underpin the key role which the lipid environment plays in modulating the rate of reaction of antioxidants characterized by different inherent chemical reactivity/membrane mobility. The accuracy of these measurements depends mainly on the pH of buffer, solvent form, temperature, AAPH/ABAP and ABTS solution preparation. Amalgamation of AAPH/ABAP and ABTS is a highly accurate combination as ABTS solution does not react with the compounds in absence of AAPH/ABAP, is not light sensitive, and does not require sophisticated techniques. On the whole, this method with kinetic analysis part is a simple way of analyzing and interpreting character and behaviour of the molecule. Altogether, a novel, facile method of study, new insights, and a quantitative understanding of the critical role in modulating peroxyl radical formation by antioxidants are reported.

## Figures and Tables

**Table 1:** List of indicator molecule with analysis system/model system for assessing peroxyl radical.

Name	Reference
Crocin	[28]
BODIPY 581/591 C11	[29]
cis-Parinaric acid	[30]
Phycoerythrin	[31]
C-phycocyanin	[32]
Dichlorofluorescin	[33]
Luminescence/chemiluminescence of luminal	[34–36]
Pyrogallol red	[37]
Analysis of lipid hydroperoxides by HPLC	[38]
Malondialdehyde-thiobarbituric assay	[39, 40]
Electron spin resonance, a spin-trapping technique	[41]
GC-MS	[42]

**Table 2:** Properties of the compound studied.

Name	Pubchem ID	Molecular formula	Molecular weight (g/mol)	XLogP3−	H-bond donor	H-bond acceptor
Trolox	4063	C_14_H_18_O_4_	250.29032	AA: 2.8	2	4
*α*-Tocopherol	14985	C_29_H_50_O_2_	430.7061	AA: 10.7	1	2
Nicotinic acid	982	C_6_H_5_NO_2_	123.11	0.4	1	3
*β* Carotene	5280489	C_40_H_56_	536.87264	13.5	0	0
Ascorbic acid	54670067	C_6_H_8_O_6_	176.12412	−1.6	4	6
Quercetin	5280343	C_15_H_10_O_7_	302.24	1.5	5	7
n-Propyl gallate	4947	C_10_H_12_O_5_	212.19928	1.8	3	5
BHA	8456	C_11_H_16_O_2_	180.24354	3.2	1	2
BHT	31404	C_15_H_24_O	220.35046	AA: 5.3	1	1
TBHQ	16043	C_10_H_14_O_2_	166.21696	2.8	2	2
tBHP	6410	C_4_H_10_O_2_	90.121	AA: 0.6	1	2
Hydrogen peroxide	784	H_2_O_2_	34.01468	−0.9	2	2

**Table 3:** IC_50_ values and lag time of the compounds in peroxyl radical averting assay.

Name	IC_50_ (µM)	Lag time (min)	Concentration
Trolox	0.119	30	10 *μ*M
*α*-Tocopherol	0.289	35	1 *μ*M
Nicotinic acid	ND	ND	ND
*β*-Carotene	91.95	15	10 *μ*M
Ascorbic acid	1.38	15	1 *μ*M
Quercetin	0.239	20	0.1 *μ*M
n-Propyl gallate	5.8	45	10 *μ*M
BHA	0.21	35	0.1 *μ*M
BHT	0.885	40	1 *μ*M
TBHQ	8.93	15	10 *μ*M
tBHP	ND	ND	ND
Hydrogen peroxide	ND	ND	ND

ND: not detected. Each data is mean average values of three independent experiments.

**Table 4:** Pearson values calculated for 10 *μ*M of various kinetic parameters.

	*V*_max⁡_	Peak	Slope	Mean	*T*: (1/2) max	Area
*V*_max⁡_	1.00	0.95	0.67	0.97	0.45	0.54
Peak	0.95	1.00	0.63	0.97	0.36	0.54
Slope	0.67	0.63	1.00	0.63	−0.01	0.81
Mean	0.97	0.97	0.63	1.00	0.39	0.54
*T*: (1/2) max	0.45	0.36	−0.01	0.39	1.00	−0.01
Area	0.54	0.54	0.81	0.54	−0.01	1.00

**Table 5:** RSQ values calculated for 10 *μ*M of various kinetic parameters.

	*V*_max⁡_	Peak	Slope	Mean	*T*: (1/2) max	Area
*V*_max⁡_	1.00	0.89	0.45	0.94	0.20	0.30
Peak	0.89	1.00	0.40	0.94	0.13	0.30
Slope	0.45	0.40	1.00	0.40	0.00	0.65
Mean	0.94	0.94	0.40	1.00	0.15	0.29
*T*: (1/2) max	0.20	0.13	0.00	0.15	1.00	0.00
Area	0.30	0.30	0.65	0.29	0.00	1.00
